# Curcumin Inhibits Membrane-Damaging Pore-Forming Function of the β-Barrel Pore-Forming Toxin *Vibrio cholerae* Cytolysin

**DOI:** 10.3389/fmicb.2021.809782

**Published:** 2022-01-24

**Authors:** Mahendra Singh, N. Rupesh, Shashi Bhushan Pandit, Kausik Chattopadhyay

**Affiliations:** Department of Biological Sciences, Indian Institute of Science Education and Research Mohali, Mohali, India

**Keywords:** pore-forming toxin, *Vibrio cholerae* cytolysin, curcumin, membranes, oligomerization

## Abstract

Vibrio cholerae cytolysin (VCC) is a β-barrel pore-forming toxin (β-PFT). Upon encountering the target cells, VCC forms heptameric β-barrel pores and permeabilizes the cell membranes. Structure-function mechanisms of VCC have been extensively studied in the past. However, the existence of any natural inhibitor for VCC has not been reported yet. In the present study, we show that curcumin can compromise the membrane-damaging activity of VCC. Curcumin is known to modulate a wide variety of biological processes and functions. However, the application of curcumin in the physiological scenario often gets limited due to its extremely poor solubility in the aqueous environment. Interestingly, we find that VCC can associate with the insoluble fraction of curcumin in the aqueous medium and thus gets separated from the solution phase. This, in turn, reduces the availability of VCC to attack the target membranes and thus blocks the membrane-damaging action of the toxin. We also observe that the soluble aqueous extract of curcumin, generated by the heat treatment, compromises the pore-forming activity of VCC. Interestingly, in the presence of such soluble extract of curcumin, VCC binds to the target membranes and forms the oligomeric assembly. However, such oligomers appear to be non-functional, devoid of the pore-forming activity. The ability of curcumin to bind to VCC and neutralize its membrane-damaging activity suggests that curcumin has the potential to act as an inhibitor of this potent bacterial β-PFT.

## Introduction

Pore-forming toxins (PFTs) are the largest class of bacterial protein toxins (Dal Peraro and van der Goot, 2016). Bacterial PFTs are generally secreted as water-soluble monomeric molecules. Upon binding to the target eukaryotic cells, bacterial PFTs form oligomeric pores in the membrane lipid bilayer that, in turn, damage the cell membranes and can eventually kill the target cells (Mondal and Chattopadhyay, 2020; Verma et al., 2021). Pore-forming proteins (PFPs) and toxins are not restricted in the pathogenic bacteria only. These membrane-damaging proteins are documented in all the domains of life (Benton and Bayly-Jones, 2021). For example, the effector functions of the immune system of the higher vertebrates including that of humans employ a number of PFPs (Krawczyk et al., 2020). The membrane-attack complex of the complement system and the perforin protein produced by the cytotoxic T-cells play critical roles in the induction of the immune functions (Liu and Lieberman, 2020; Spicer and Dunstone, 2021). Gasdermin D is another distinct PFP that plays crucial roles in the inflammatory cell death or pyroptosis in the mammalian system (Lieberman et al., 2019). PFTs and PFPs from the diverse kingdoms of life share very little or no similarity in their amino acid sequences, and they highlight enormous variations in their structures and mechanisms of actions (Mondal et al., 2020).

*Vibrio cholerae* cytolysin (VCC) is a potent membrane-damaging PFT secreted by the Gram-negative bacterial pathogen *V. cholerae*, the causative agent of the severe diarrheal disease Cholera (Honda and Finkelstein, 1979; Yamamoto et al., 1984; De and Olson, 2011). VCC exhibits potent cytolytic/cytotoxic activity against a wide range of eukaryotic cells that include erythrocytes, intestinal epithelial cells, as well as immune cells (McCardell et al., 1999; Saka et al., 2008; Khilwani and Chattopadhyay, 2015). VCC also exerts enterotoxic activity in the ligated rabbit ileal loop model of the diarrheal disease (Ichinose et al., 1987). Apart from the membrane-damaging pore-forming functionality, VCC can also initiate a plethora of signaling pathways leading to the diverse cellular responses that include autophagy as well as programmed apoptotic cell death (Gutierrez et al., 2007; Saka et al., 2008). It has also been reported that VCC has the potential to contribute toward the diarrhea and cholera-like symptoms, particularly caused by those strains that cannot produce the Cholera Toxin (Saka et al., 2008). Altogether, these pathophysiological properties exhibited by VCC enable it to serve as a potent virulence factor of *V. cholerae*.

The mature active form of VCC is a monomeric ∼65 kDa protein. Upon interacting with the target eukaryotic cell membranes, or cholesterol-containing membrane lipid bilayer, it assembles into heptameric transmembrane β-barrel pores (De and Olson, 2011; Kathuria et al., 2018). Specific binding of VCC with membrane phospholipids and cholesterol regulates the efficacy of its membrane-binding step (Rai and Chattopadhyay, 2015; Kathuria et al., 2018). In addition, VCC employs a specific lectin-like activity to recognize and bind to the cell-surface glycans present in the biomembranes (Rai et al., 2013). Upon binding to the target membranes, VCC monomers assemble into the pre-pore oligomeric intermediates (Paul and Chattopadhyay, 2014). Subsequently, pore-forming motifs from the toxin protomers insert into the membranes to generate the functional transmembrane pores (Rai and Chattopadhyay, 2014).

The membrane pore-formation mechanism of the PFTs and their functional consequences in the context of the bacterial pathogenesis processes are being studied for more than the past three decades. In the same direction, structure-function mechanisms of VCC have also been studied extensively in the past. However, limited information is available regarding the usage of any natural inhibitor that can potentially block the membrane-damaging pore-forming action of the PFTs, including that of VCC. Pathogenic bacteria tend to develop antibiotic resistance. Therefore, natural inhibitors appear to be the more promising candidates to directly target the virulence factors in the pathogenic microbes. Accessory virulence factors like VCC are not generally essential for the survival of the pathogen. Therefore, application of the natural inhibitors targeting the specific virulence factors, rather than using antibiotics, is a promising alternate approach to combat the bacterial virulence mechanisms.

Curcumin is a small polyphenolic compound extracted from the rhizomes of turmeric (*Curcuma longa*) (Priyadarsini, 2014). Curcumin has been shown to possess a wide range of biological and pharmacological properties that include anti-bacterial, anti-inflammatory, and anti-oxidant activities (Schraufstatter and Bernt, 1949; Amalraj et al., 2017; Sharifi-Rad et al., 2020). Curcumin has also been shown to neutralize the membrane-damaging action of some of the bacterial PFTs, such as Listeriolysin O (LLO) produced by *Listeria monocytogenes* and α-hemolysin secreted by *Staphylococcus aureus* (Wang et al., 2016; Zhou et al., 2017). In both cases, curcumin has been shown to bind to the toxins and hinder their self-assembly toward oligomeric pore-formation (Wang et al., 2016; Zhou et al., 2017).

In the present study, we report that curcumin has the ability to block the pore-forming activity of VCC. To examine the inhibitory effect of curcumin on VCC, we have used two different curcumin preparations. In one case, curcumin, dissolved in the organic solvent, has been introduced to VCC in the aqueous medium. Due to its very low solubility in the aqueous medium, curcumin becomes insoluble upon such treatment. We have observed that VCC tends to associate with the insoluble fraction of curcumin. This reduces the availability of VCC in the solution for executing its membrane-damaging pore-forming effect on its target membranes. In another strategy, soluble aqueous extract of curcumin has been prepared by heat treatment. We have observed that such a soluble extract of curcumin in the aqueous solution, obtained after heat treatment, can also inhibit the pore-forming activity of VCC. However, the presence of the soluble aqueous extract of curcumin does not interfere with the binding propensity of VCC toward the target membranes. Also, in the presence of the soluble aqueous extract of curcumin, VCC retains its ability to form the oligomeric assembly in the target membranes. These observations suggest that the pore-forming function of VCC gets inhibited by two different means, depending on the mode of the curcumin preparation. Taken together, our study for the first time shows the efficacy of curcumin as a potent inhibitor for the membrane-damaging pore-forming activity of VCC.

## Materials and Methods

### Ethics Statement

The experiment using human blood was approved by the Institute Ethics Committee of Indian Institute of Science Education and Research Mohali. Written informed consent was obtained from each donor.

### Purification of *Vibrio cholerae* Cytolysin

*Vibrio cholerae* cytolysin was expressed and purified following the methods described in the earlier studies (Olson and Gouaux, 2005; Paul and Chattopadhyay, 2012; Mondal et al., 2021). Briefly, the nucleotide construct encoding the precursor form of VCC (pro-VCC; corresponding to the NCBI Reference Sequence WP_001125271.1 and the UniProt ID A0A0H6SZL4_VIBCL) cloned into the pET-14b vector was transformed into the *Escherichia coli* Origami B cells. Protein over-expression was induced with 1 mM IPTG at 30°C for 3 h. The soluble form of His-tagged pro-VCC was purified from the bacterial cell lysate using the Ni-NTA Agarose (Qiagen) affinity chromatography, followed by ion-exchange chromatography on Q Sepharose Fast Flow resin (Merck) (Supplementary Figure 1). The mature form of VCC was generated by treatment with trypsin using protein:trypsin ratio of 2000:1, for 5 min at 25°C. Mature VCC was subjected to another round of purification using Q Sepharose Fast Flow anion-exchange chromatography. The purity of the protein was confirmed by SDS-PAGE/Coomassie staining (Supplementary Figure 1). Molecular weight of the purified protein was confirmed with respect to the protein molecular weight marker [PageRuler™ Prestained Protein Ladder, 10–180 kDa, from Thermo Fisher Scientific (Catalog number: 26616)]. Protein concentration was determined by measuring absorbance at 280 nm, using the theoretical extinction coefficient predicted from the primary structure.

### Preparation of Curcumin Solutions

Curcumin solution in dimethyl sulfoxide (DMSO) (curcumin-DMSO) was prepared by dissolving 20 mg of curcumin powder (Merck) into 1 ml DMSO, unless mentioned otherwise. The solution was mixed thoroughly and filtered through 0.22 μm syringe filter. As the curcumin preparation was completely soluble in DMSO, concentrations in all the reaction mixtures were expressed in the form of weight/volume (calculated based on the weight of the curcumin powder taken to prepare the stock solution in DMSO).

Curcumin is poorly soluble in the aqueous medium. However, it has been shown in an earlier report that the solubility of curcumin in the aqueous medium can be increased upon heating (by incubating at 90°C, followed by incubation in the boiling water bath) (Kurien et al., 2007). It has also been shown in that study that the heat-extraction in the aqueous medium does not lead to any change in the curcumin structure due to any heat-induced disintegration of the curcumin molecule. Based on such report, we also prepared the soluble extract of curcumin in the aqueous medium by heating. For the preparation of the soluble extract of curcumin in the aqueous buffer, 10 mg curcumin powder was added into 2 ml of PBS (10 mM Na_2_HPO_4_, 1.8 mM KH_2_PO_4_, 137 mM NaCl, 2.7 mM KCl, pH 7.4) (curcumin-PBS), or TBS (20 mM Tris-HCl, 150 mM NaCl, pH 8.0) (curcumin-TBS), unless mentioned otherwise. The mixture was vortexed and subjected to heating at 99°C in a water bath for 1 h. Due to the poor solubility in the aqueous medium, the majority of the material remained insoluble even after the heat extraction in the aqueous buffer. Therefore, the preparation was centrifuged at 17,000 × *g* for 45 min. The supernatant was collected, filtered through a 0.22 μm syringe filter, and was used for performing the assays. In all the assays, the amount of the aqueous extract of curcumin present in the reaction mixture was represented in the form of %volume/volume of the extract (using the volume of the extract/total reaction volume).

### Mass Spectrometric Analysis of the Curcumin Preparations

Mass spectrometric analyses were performed for the curcumin preparations, either dissolved in the organic solvent DMSO, or generated upon heat extraction in the aqueous medium in water. Final concentration of curcumin in DMSO was 20 μg/ml. The aqueous extract of curcumin was prepared in water by the heat treatment, as described above, from a suspension of 10 mg curcumin powder in 2 ml water. Aqueous extract of curcumin was prepared in water to avoid interference by the buffer salts during mass spectrometry. 50 μl of each of these preparations were dissolved in 950 μl of the make-up phase (Acetonitrile and 0.1% formic acid). Samples were analyzed on a SYNAPT G2-Si High Definition Mass Spectrometer with a Q-TOF analyzer (Waters). Sample solutions were infused into the electrospray ionization source, operating at the negative ion mode, at a flow rate of 0.2 ml/min, with the mobile phase consisting of 20% aqueous (0.1% formic acid in water) and 80% organic (0.1% formic acid in acetonitrile) solvent. The electrospray voltage was set to 2 kV, and the sample cone voltage was 40 volts. The source temperature was 120°C, and the de-solvation temperature was 300°C. Nitrogen was used as the cone gas, de-solvation gas, and nebulizer gas, at the flow rates of 60 lh^–1^, 600 lh^–1^ and 6 bar, respectively. The mass range for the acquisition was from 50 to 1,500. The data were collected and analyzed with the MassLynx V4.1 software. Data were shown for the m/z values in the range of 200–500.

Curcumin preparations were subjected to negative mode electrospray ionization mass spectrometry, and the corresponding mass spectra were acquired (Supplementary Figure 2). For curcumin dissolved in DMSO, the peak corresponding to *m/z* 367.12 was found to be the base peak, presumably representing the greatest relative abundance (100%) of the deprotonated state of curcumin, as the molecular weight of intact curcumin is 368.38. A small peak at *m/z* 368.12 represented the intact curcumin molecule without deprotonation. Mass spectrometry profile of the aqueous extract of curcumin, generated upon heating, showed that the peak intensity at *m/z* 367.12 was highly reduced, and the additional *m/z* peaks became more prominent. A substantially reduced peak intensity at *m/z* 367.12 is presumably due to the fact that curcumin is scarcely soluble in the aqueous medium.

### Assay of Hemolytic Activity

Hemolytic activity of VCC (either pre-treated with curcumin-DMSO preparation or pre-incubated in the curcumin-PBS extract) against human erythrocytes was measured following the methods described earlier (Mondal et al., 2021). Briefly, human erythrocytes were washed with PBS and were added to VCC (with or without curcumin treatment) in PBS to a final reaction volume of 1 ml. Erythrocyte concentration was adjusted in the final reaction volume corresponding to the optical density of ∼0.9–1 at 650 nm (OD_650_). Concentrations of VCC and curcumin, described in the Results section, represented the final concentrations in the final 1 ml reaction volume. Hemolytic activity of VCC (with or without curcumin treatment) against the human erythrocytes was monitored by measuring the decrease in the turbidity (OD_650_) of the reaction mixture, spectrophotometrically. Treatment with 100 nM VCC, in the absence of curcumin, resulted in the complete lysis of the human erythrocytes, thus corresponding to the 100% hemolytic activity.

### Calcein-Release Assay of Pore-Formation in the Membrane Lipid Bilayer of Liposomes

Pore-forming activity of VCC in the membrane lipid bilayer of lipid vesicles or liposomes was monitored by measuring the release of calcein from the liposomes upon treatment with the protein. Asolectin-cholesterol liposomes [1:1 weight ratio of Asolectin:cholesterol; with 1 mg/ml final lipid concentration in 20 mM Tris-HCl, 150 mM NaCl, pH 8.0 (TBS)] containing calcein was prepared following the method described earlier (Paul and Chattopadhyay, 2012). 25 μl of the liposome preparation was added to VCC in the presence or absence of the curcumin extract in TBS in a reaction volume of 2 ml. Prior to the addition of the liposomes, VCC was pre-incubated in the presence of the curcumin extract for 30 min at room temperature. Release of calcein from the liposomes upon membrane pore-formation by VCC was monitored by measuring fluorescence at 520 nm upon excitation at 488 nm. Treatment of the liposomes with 6 mM sodium deoxycholate corresponded to 100% calcein release, while the treatment with buffer or the buffer containing curcumin extract without protein was taken as the corresponding negative control.

### Tryptophan Fluorescence Spectroscopy

Intrinsic tryptophan fluorescence of VCC (200 nM) was monitored either in PBS upon treatment with curcumin from a stock solution in DMSO (10 mg/ml stock solution of curcumin dissolved in DMSO), or in the presence of curcumin extract prepared in PBS, in a reaction volume of 2 ml. Fluorescence emission spectra were collected on a FluoroMax-4 (Horiba) spectrofluorimeter set at 25°C, after a pre-incubation of 30 s, upon excitation at 295 nm. All the spectra were corrected for the corresponding control spectra without protein. Data shown here are the normalized fluorescence intensity values with respect to the maximal fluorescence intensity value in each data set.

The dissociation constant (*K*_D_) of the VCC-curcumin interaction was determined by non-linear regression of the fluorescence data (relative decrease in the tryptophan fluorescence intensity at 339 nm in the presence of curcumin) plotted against the curcumin concentration (in μM) using the hyperbola function [*y* = P1*x/(P2 + *x*); *y* = relative decrease in the tryptophan fluorescence intensity at 339 nm, *x* = curcumin concentration in μM; P2 determined from the expression corresponds to the *K*_D_ value] in the program OriginPro. Relative decrease in the normalized tryptophan fluorescence intensity (at 339 nm) was determined using the expression (*F*_o_ − *F*_curcumin_)/*F*_o_, in which *F*_o_ is the normalized fluorescence intensity at 339 nm in the absence of curcumin, and *F*_curcumin_ represents the normalized fluorescence intensity at 339 nm in the presence of a specific concentration of curcumin.

### Flow Cytometry-Based Assay of Binding With Erythrocytes

The binding of VCC with human erythrocytes in the presence of curcumin extract in PBS was monitored using the flow cytometry-based assay, as described previously (Paul and Chattopadhyay, 2012). Briefly, VCC (75 nM) was pre-incubated in the absence or presence of the curcumin extract in PBS [80% (vol/vol)] for 30 min at room temperature. Subsequently, human erythrocytes (10^6^ cells) were added to the reaction mixture in 100 μl volume, and incubated at 4°C for 30 min. Incubation at such low temperature allows binding of VCC to the erythrocytes but blocks oligomeric pore-formation and lysis of the cells, thus enabling flow cytometry-based detection of the erythrocytes-bound VCC. Cells were subsequently stained with anti-VCC antisera by incubating at 4°C, followed by staining with FITC-labeled secondary antibody at 4°C. Cells were acquired on a FACSCalibur (BD Biosciences) flow cytometer, and data were analyzed with the FlowJo software. Cells without VCC treatment but stained with primary and secondary antibodies served as the control.

### Pull Down-Based Assay With Human Erythrocytes Membrane Ghosts

For the preparation of human erythrocytes membrane ghost, human blood (3 ml) was washed repeatedly with PBS by centrifugation at 800 × *g*, and then re-suspended in 50 ml ice-cold hypotonic lysis buffer (4 mM Na_2_HPO_4_.2H_2_O, 1 mM NaH_2_PO_4_, 1 mM EDTA, pH 7.5). After incubation for 30 min in ice, it was subjected to centrifugation at 15,000 × *g* for 30 min. The pellet fraction was collected and washed repeatedly with the hypotonic lysis buffer till it was free of any unlysed cell. The pellet fraction containing the erythrocytes membrane ghost was then re-suspended in 50 ml 10 mM sodium phosphate buffer, containing 5 mM MgCl_2_ (pH 7.5), and incubated at 37°C for 40 min. After incubation, the suspension was centrifuged at 15,000 × *g* for 30 min, and the pellet was re-suspended in 3 ml 10 mM sodium phosphate buffer, containing 5 mM MgCl_2_ (pH 7.5), and mixed thoroughly. The protein concentration in the human erythrocytes membrane ghost preparation was estimated by standard Bradford assay and was found to be ∼0.48 mg/ml.

SDS-stable oligomer formation by VCC in the human erythrocytes membrane ghost, in the absence or presence of the curcumin extract in PBS, was examined by pull down-based assay followed by immunoblotting. For this, 90 μl human erythrocytes membrane ghost preparation was added to VCC in the absence or presence of curcumin extract in PBS (90% vol/vol), in a reaction volume of 1 ml in PBS. The final concentration of VCC in the reaction mixture was adjusted to 100 nM. After incubation at room temperature for 60 min, the reaction mixtures were subjected to ultracentrifugation for 30 min at 1,05,000 × *g*. The pellet fractions were washed once with PBS and re-suspended in 40 μl PBS. Each of the pellet fractions was divided into two equal parts, to which SDS-PAGE sample buffer was added. One part was boiled for 30 min, while the other part was incubated at 30°C. Samples were then subjected to SDS-PAGE followed by immunoblotting with anti-VCC antiserum and HRP-conjugated secondary antibody. Immunoblots were developed using the Clarity Western ECL Substrate (Bio-Rad), and images were acquired on ImageQuant LAS4000 (GE Healthcare Life Sciences).

### Pull Down-Based Assay to Examine the Binding of *Vibrio cholerae* Cytolysin With Curcumin

Pull down-based assay was used to examine the association of VCC with the insoluble fraction of curcumin in the aqueous buffer. For this, 25 μl from a stock solution of curcumin dissolved in DMSO (20 mg/ml) was added to VCC (at final concentrations of 0.25, 0.5, 1, and 2 μM) in a final reaction volume of 1 ml in PBS. Upon addition to the aqueous buffer, curcumin formed insoluble particles. After 1 h incubation at room temperature, the reaction mixtures were subjected to centrifugation at 17,000 × *g* for 45 min. The supernatant and the pellet fractions were collected separately. The pellet fractions were re-suspended in 1 ml PBS and mixed thoroughly. Aliquots of 20 μl from both the supernatant and pellet fractions were boiled with SDS-PAGE sample buffer and were subjected to SDS-PAGE/Coomassie staining to detect the presence of VCC.

We also examined whether VCC could form SDS-stable oligomers upon association with the insoluble particles of curcumin. For this, 25 μl from a stock solution of curcumin dissolved in DMSO (20 mg/ml) was added to VCC (4 μM) in a final reaction volume of 1 ml in PBS, and incubated for 60 min at room temperature. 40 μl of the reaction mixture was withdrawn for use as a control input, and the rest was then subjected to centrifugation at 10,000 rpm for 10 min. The supernatant was removed, and the pellet fraction was re-suspended in 40 μl PBS. 40 μl of the input control, supernatant, and the re-suspended pellet fraction were divided into two equal parts. One part was boiled with SDS-PAGE sample buffer, while the other part was kept unboiled in the presence of the SDS-PAGE sample buffer. Samples were then analyzed by SDS-PAGE/Coomassie staining. Unboiled samples allowed detection of the SDS-stable oligomers of VCC, if formed. As a control, 25 μl DMSO was added to VCC (4 μM) in a reaction volume of 1 ml, and incubated for 60 min at room temperature. Under such condition, VCC did not form any SDS-stable oligomer to any noticeable extent, thus negating any effect of DMSO alone.

### Transmission Electron Microscopy to Examine the Assembly State of *Vibrio cholerae* Cytolysin Upon Association With Curcumin

Transmission electron microscopy (TEM) was used to visualize the assembly state of VCC upon association with the insoluble fraction of curcumin in the aqueous buffer. For this, 10 μl from a stock solution of curcumin dissolved in DMSO (20 mg/ml) was added to VCC (2 μM) in a final reaction volume of 1 ml in PBS, and was incubated for 1 h at room temperature. For the control reaction, 20 μl from a stock solution of curcumin dissolved in DMSO (20 mg/ml) was added to the reaction volume of 1 ml in PBS without VCC. The reaction mixtures were subjected to centrifugation at 10,000 rpm for 10 min. The pellets were re-suspended in 200 μl PBS. Samples (4μl) were added to the plasma cleaned 400 mesh copper grids with continuous carbon support (Aritech Chemazone Pvt. Limited), and incubated for 1 min. The excess amount of sample was blotted off and samples were negatively stained with 2% uranyl acetate for 1 min. The excess uranyl acetate was blotted off and the samples were dried at room temperature. The samples were analyzed on a JEM-F200 electron microscope (Jeol Inc.) operating at 200 kV, and images were recorded with a Gatan OneView camera (Gatan Inc.) at a nominal magnification of 60,000×, with a defocus −2.5 μm and the cumulative fluence for the images was limited to ∼60 electrons/pixel.

### Pull Down-Based Assay to Monitor Binding and SDS-Stable Oligomer Formation by *Vibrio cholerae* Cytolysin in the Liposomes in the Presence of Curcumin Extract in Aqueous Buffer

Pull down-based assay was performed to examine binding and SDS-stable oligomer formation by VCC in the membrane lipid bilayer of the liposomes in the presence of curcumin extract in PBS. Asolectin-cholesterol liposomes (1:1 weight ratio of Asolectin and cholesterol; total lipid concentration of 1 mg/ml in PBS) were prepared following the method described earlier (Paul and Chattopadhyay, 2012). VCC (500 nM) was pre-incubated with the curcumin extract in PBS (90% vol/vol) for 30 min at room temperature. Asolectin-cholesterol liposomes (65 μl) were added to this reaction mixture in a final reaction volume of 1 ml, and incubated at 25°C for 1 h. The reaction mixtures were subjected to ultra-centrifugation at 1,05,000 × *g* for 30 min, the pellet fractions were collected and washed twice with PBS, and re-suspended in 40 μl PBS. Re-suspended pellet fractions were divided into two equal parts and mixed with the SDS-PAGE sample buffer. One part was boiled, and the other part was kept at room temperature. Samples were subjected to SDS-PAGE followed by Coomassie staining. The unboiled samples allowed detection of SDS-stable oligomeric bands formed by VCC, if any. VCC treated with the liposomes in the absence of curcumin extract served as the control.

### Prediction of the Ligand-Binding Site and Docking of Curcumin Onto the *Vibrio cholerae* Cytolysin Structure

We have used the energy-minimized VCC structure from our previous work (Mondal et al., 2021) for the prediction of the binding sites and docking studies. The binding pockets were predicted using the machine-learning methods [DeepSite (Jimenez et al., 2017) and P2rank (Krivak and Hoksza, 2018)] and CurPocket (Cao and Li, 2014; Liu et al., 2020), which detect cavities based on the curvature of the protein surface. The consensus of the predicted pocket centers was obtained by clustering sites, which are within the 5 Å distances of each other.

The three-dimensional structure of the keto-form of curcumin (ligand ID: CC9) was extracted from human DYRK2 bound to curcumin [Protein Data Bank (PDB) ID: 6HDR]. The protein and the ligand for docking were prepared using the command line tools of the AutoDock 4.2.6 program. The docking was performed using Vina 1.1.2 (Trott and Olson, 2010) with the predicted binding site centers and flexible ligand as inputs to the program. The ligand search box was cubic with the dimension of 27 Å as the side. We performed docking on all the predicted binding sites and ranked the docked poses based on the docking energy score. The details of the protein-ligand interactions are generated using LigPlot+.

### Visualization of the Structural Models

Structural models were visualized using PyMOL (The PyMOL Molecular Graphics System, Version 2.5.0 Schrödinger, LLC.), or VMD (Humphrey et al., 1996). Protein structural coordinates of the monomeric (PDB ID: 1XEZ) and oligomeric form of VCC (PDB ID: 3O44) were retrieved from the PDB.

## Results and Discussion

### Curcumin Compromises the Pore-Forming Hemolytic Activity of *Vibrio cholerae* Cytolysin

**FIGURE 1:**
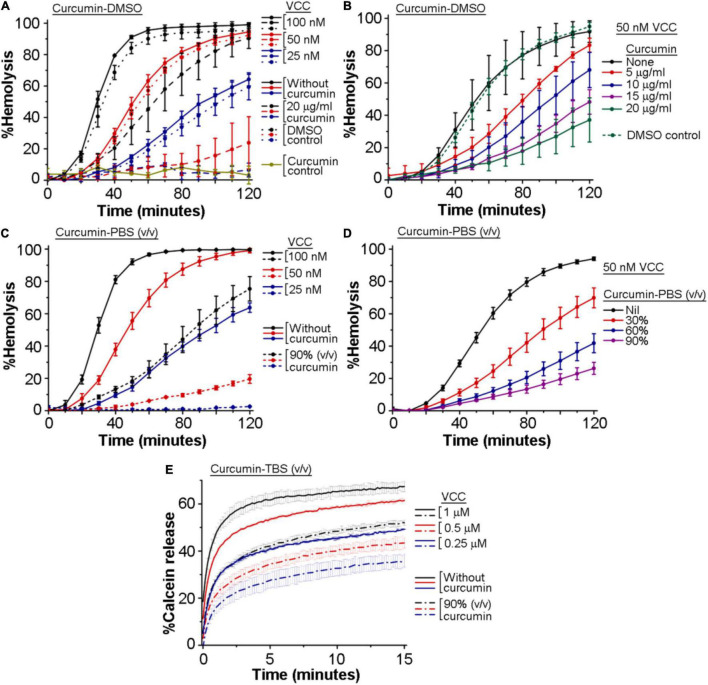
Curcumin compromises the membrane-damaging pore-forming activity of *Vibrio cholerae* cytolysin (VCC). **(A–D)** Curcumin inhibits the pore-forming hemolytic activity of VCC against the human erythrocytes. **(A,B)** Hemolytic activity of VCC upon treatment with curcumin from a stock solution prepared in DMSO (curcumin-DMSO). As shown in **(A)**, equivalent volumes of DMSO without curcumin (denoted as the DMSO control in **A**) did not affect the hemolytic activity of VCC. Also in **(B)**, equivalent volume of DMSO without curcumin (volume of DMSO corresponding to the treatment with 20 μg/ml curcumin-DMSO) (designated as the DMSO control in **B**) did not affect the hemolytic activity of VCC to any noticeable extent. Presence of 20 μg/ml curcumin (added from a stock solution in DMSO) did not induce any lysis of the erythrocytes in the absence of VCC, to any noticeable extent (shown as the curcumin control in **A**), suggesting that curcumin and DMSO alone do not affect the erythrocytes in the absence of the VCC treatment. **(C,D)** Hemolytic activity of VCC in the presence of the curcumin extract in PBS (curcumin-PBS). VCC was pre-incubated with the curcumin preparations at room temperature for 30 min before the addition of the erythrocytes. **(E)** Curcumin inhibits the pore-forming activity of the VCC against the membrane lipid bilayer of the Asolectin-cholesterol liposomes. VCC was pre-incubated in the presence of the curcumin extract in TBS (curcumin-TBS), at room temperature for 30 min, before the addition of the liposomes. Concentrations of VCC and curcumin shown here represent the final amount present in the reaction mixture during the hemolytic activity and the calcein-release assay. Data shown here are the averages ± standard deviations of three independent treatments.

We wanted to examine whether curcumin has any inhibitory effect on the pore-forming activity of VCC. For this, we monitored the inhibitory effect of curcumin, if any, on the hemolytic activity of VCC against the human erythrocytes. We employed two different preparations of curcumin to study its effect on the VCC-mediated hemolytic activity. In one case, VCC, in PBS, was treated with curcumin, where curcumin was added from a stock preparation solubilized in DMSO (designated as curcumin-DMSO). In another case, VCC was incubated with the soluble extract of curcumin, obtained by heat-treatment in PBS (designated as curcumin-PBS). We observed that both the preparations of curcumin compromised the pore-forming hemolytic activity of VCC against the human erythrocytes, when tested over a range of protein concentrations, as well as when examined with varying amounts of curcumin (Figures 1A,D). For example, 20 μg/ml of curcumin-DMSO resulted in ∼50–70% inhibition of the hemolytic activity of VCC, when tested for the protein concentration of 50 nM, over a time period of 2 h (Figures 1A,B). Similarly, 50 nM VCC showed ∼70–80% reduction in the hemolytic activity in the presence of 90% (volume/volume) curcumin-PBS [heat extracted from curcumin (5 mg) suspension in PBS (1 ml)], when monitored over a time period of 2 h (Figures 1C,D). Altogether, these results suggest that curcumin has the ability to block the pore-forming hemolytic activity of VCC against the human erythrocytes.

### Curcumin Compromises the Pore-Forming Activity of *Vibrio cholerae* Cytolysin in the Membrane Lipid Bilayer of Liposomes

We examined the inhibitory effect of curcumin on the pore-forming activity of VCC against the membrane lipid bilayer of liposomes constituted with Asolectin and cholesterol (50% weight ratio). Pore-forming activity of VCC was estimated by monitoring the release of calcein from the Asolectin-cholesterol liposomes upon treatment with the toxin (using three different protein concentrations of 1, 0.5, and 0.25 μM). We observed that in the presence of the curcumin-PBS extract (90%, volume/volume), the extent of calcein-release triggered by VCC was noticeably compromised at all the three toxin concentrations (Figure 1E). The effect of curcumin-DMSO could not be examined using this assay, as this preparation of curcumin was found to interfere with the calcein fluorescence to a significant extent. Altogether, our data suggest that curcumin compromises the pore-forming activity of VCC against the membrane lipid bilayer of Asolectin-cholesterol liposomes.

### Intrinsic Tryptophan Fluorescence of *Vibrio cholerae* Cytolysin Gets Quenched in the Presence of Curcumin

**FIGURE 2:**
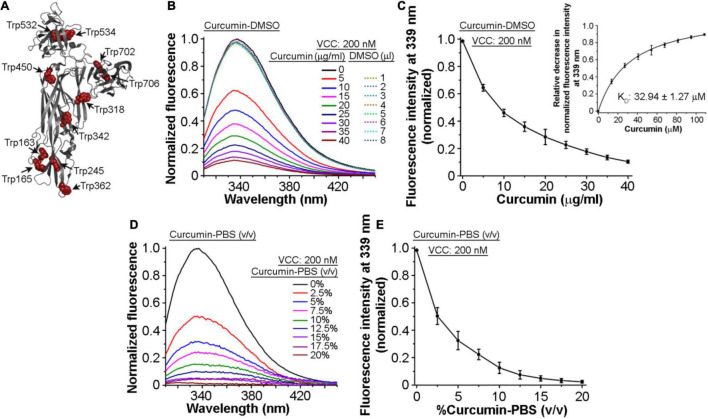
Intrinsic tryptophan fluorescence of VCC gets quenched in the presence of curcumin. **(A)** Structural model of VCC showing the distribution of the tryptophan residues throughout the protein structure. **(B,C)** Intrinsic tryptophan fluorescence emission profile of VCC upon treatment with curcumin from a stock solution prepared in DMSO (curcumin-DMSO). Equivalent volumes of DMSO without curcumin did not affect the tryptophan fluorescence emission profile of VCC (shown with the dotted curves in **B**). Non-linear fitting of the fluorescence data (relative decrease in the tryptophan fluorescence at 339 nm) against the varying concentration of curcumin revealed the *K*_D_ value of the VCC-curcumin interaction in the range of ∼32.94 μM (inset in **C**). **(D,E)** Intrinsic tryptophan fluorescence emission profile of VCC in the presence of the curcumin extract in PBS (curcumin-PBS). Data shown in **(B,D)** are the representatives of four independent experiments. Data shown in **(C,E)** are the averages ± standard deviations of four independent experiments. Concentrations of VCC and curcumin shown here represent the final amount present in the reaction mixture.

Toward exploring the mechanism of the inhibitory effect of curcumin on the pore-forming activity of VCC, we examined whether VCC could interact with curcumin. For this, we monitored the tryptophan fluorescence emission profile of VCC in the presence of curcumin. Mature form of VCC harbors 11 tryptophan residues that are distributed throughout the protein structure (Figure 2A). Any change in the tryptophan fluorescence emission in the presence of curcumin would suggest a direct interaction of the protein with curcumin. We observed that the addition of an increasing amount of curcumin-DMSO resulted in the prominent decrease in the tryptophan emission fluorescence of VCC in a progressive manner (Figures 2B,C). With the increasing concentration of curcumin, we also observed a marginal ∼1–2 nm blue-shift in the wavelength maximum of the tryptophan fluorescence. This is consistent with the fact that upon interaction with curcumin, surface-exposed tryptophan residues would be expected to be quenched more as compared to those buried within the protein structure. Therefore, under the condition of the quenched state(s) of the surface-exposed tryptophan residues in the presence of curcumin, buried tryptophan residues would contribute more toward the fluorescence emission, thus showing an overall blue-shifted emission maximum. We determined the *K*_D_ value of the VCC-curcumin interaction from the tryptophan fluorescence emission profile of VCC in the presence of the varying curcumin concentration. Non-linear fitting of the fluorescence data (relative decrease in the tryptophan fluorescence at 339 nm) against the varying concentration of curcumin allowed us to obtain the *K*_D_ value in the range of ∼32.94 μM (Figure 2C, Inset). The tryptophan fluorescence emission of VCC was also found to be decreased progressively, when monitored in the presence of an increasing amount of curcumin-PBS extract (Figures 2D,E). Altogether, these results indicated that curcumin could bind to VCC and quench the intrinsic tryptophan fluorescence emission of the protein.

### *Vibrio cholerae* Cytolysin Tends to Associate With the Insoluble Fraction of Curcumin and Forms SDS-Stable Oligomeric Assembly

**FIGURE 3:**
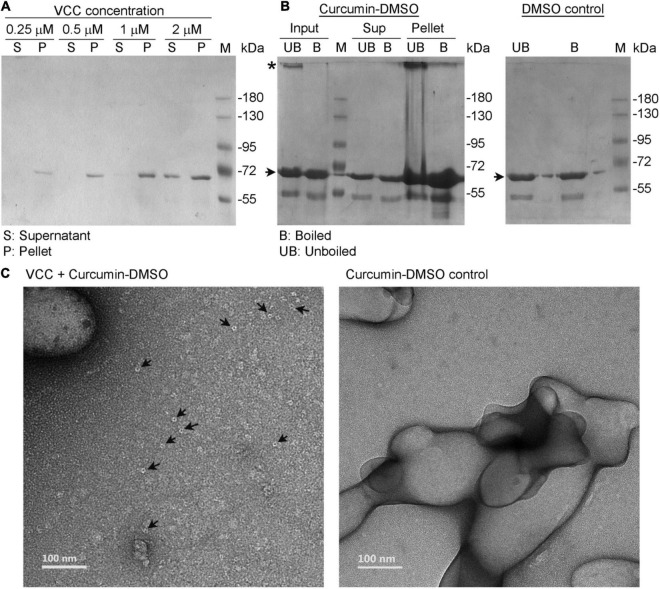
*Vibrio cholerae* cytolysin tends to associate with the insoluble fraction of curcumin and forms SDS-stable oligomers. **(A)** Curcumin, from a stock solution prepared in DMSO, was added to VCC (at four different concentrations; 0.25, 0.5, 1, and 2 μM) in the aqueous buffer in PBS. The final concentration of curcumin in the reaction mixture was adjusted to 500 μg/ml. Association of VCC with the insoluble fraction of curcumin, generated upon exposure to the aqueous environment, was probed by the pull down-based assay, in which pellet and the supernatant fractions were analyzed by SDS-PAGE and Coomassie staining. **(B)** Curcumin, from a stock solution prepared in DMSO, was added to VCC (4 μM) in the aqueous buffer in PBS, and the final concentration of curcumin was adjusted to 500 μg/ml. SDS-stable oligomer formation by VCC associated with the insoluble fraction of curcumin was probed by pull down-based assay, in which the pellet and the supernatant fractions were analyzed by SDS-PAGE and Coomassie staining, with or without boiling in the SDS-PAGE sample buffer. Unboiled fractions allowed detection of the SDS-stable oligomer formation by VCC, if any. For a control reaction, equivalent volume of DMSO, lacking curcumin, was also added to VCC (right panel). The bands corresponding to the monomeric and oligomeric form of VCC are indicated with arrow and asterisk, respectively. Lanes marked as M show the protein molecular weight markers. Data shown here are the representatives of three independent experiments. **(C)** TEM-based imaging showed formation of some ring-like structure by VCC (indicated with arrow) upon association with the insoluble fraction of curcumin (left panel). Such structures were not documented in the curcumin control without VCC (right panel).

To monitor the direct association of VCC with curcumin, we also employed a pull down-based assay. For this, curcumin-DMSO (to a final concentration of 500 μg/ml in the reaction mixture; added from a stock solution of 20 mg/ml curcumin in DMSO) was added to VCC (varying concentrations of 0.25, 0.5, 1, and 2 μM) in PBS. The reaction mixtures were incubated for 1 h at room temperature, and then subjected to centrifugation to allow precipitation of the insoluble fraction of the curcumin generated upon exposure to the aqueous buffer. Subsequently, the pellet fractions were probed for the presence of any bound VCC by SDS-PAGE and Coomassie staining. We observed that VCC, over the range of protein concentrations used in this assay, showed a prominent propensity to associate with the insoluble pellet fraction of curcumin (Figure 3A). Therefore, this result confirmed the notion that VCC is indeed capable of binding with curcumin. This, in turn, also explains the inhibitory effect of curcumin-DMSO on the pore-forming activity of VCC. Upon addition of the curcumin-DMSO preparation into the aqueous buffer containing VCC, curcumin tends to become insoluble. As a result, VCC also tends to partition to the insoluble fraction of the curcumin, presumably due to its prominent binding propensity toward curcumin. Therefore, the availability of VCC in solution for exerting the pore-forming activity on the target membranes gets reduced.

Being a prominent member in the family of β-PFTs, VCC forms oligomeric pores in the target membranes. Such oligomeric pore assembly displays remarkable stability and can be visualized as oligomeric bands in the SDS-PAGE/Coomassie staining, when probed under the unboiled condition. Interestingly, VCC has been shown to form SDS-stable oligomeric assembly upon association with cholesterol, even in the absence of the membrane lipid bilayer (Harris et al., 2002). In the present study, we examined whether VCC could form a similar SDS-stable oligomeric assembly upon binding to the insoluble fraction of curcumin. We employed the pull down-based assay to isolate VCC associated with the insoluble fraction of curcumin and examined by SDS-PAGE/Coomassie staining without boiling. Our result showed that a noticeable fraction of VCC associated with insoluble curcumin migrated as an SDS-stable oligomeric band under the unboiled condition in the SDS-PAGE (Figure 3B). Such SDS-stable oligomer of VCC was not detected to any noticeable extent in the presence of DMSO only (lacking curcumin), thus negating any effect of DMSO alone (Figure 3B, right panel). These data suggest that VCC has the propensity to associate with the insoluble fraction of curcumin in suspension, and upon association, VCC could form SDS-stable oligomeric assembly. It is possible that the insoluble curcumin fraction provides a binding platform for VCC, association with which enhances the local concentration of the toxin. This, in turn, can favor the propensity of VCC to form the SDS-stable oligomeric assembly, which is not otherwise formed in the solution phase. It is interesting to note that the TEM-based imaging also showed formation of some ring-like structures by VCC upon association with the insoluble fraction of curcumin (Figure 3C). Similar ring-like pore structures are typically formed by VCC in the lipid bilayer of the target membranes (Mondal et al., 2021). However, it remains unclear at present whether such oligomeric assembly of VCC is similar to the pore states of the protein formed in the membranes, or it represents an off-pathway abortive assembly state of the toxin.

### *Vibrio cholerae* Cytolysin Retains Its Ability to Associate With the Target Membranes and Form SDS-Stable Oligomeric Assembly in the Presence of the Soluble Aqueous Extract of Curcumin

**FIGURE 4:**
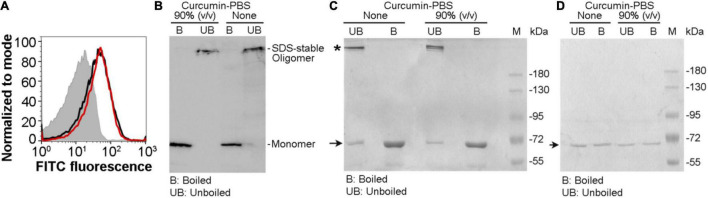
*Vibrio cholerae* cytolysin retains its ability to bind to the target membranes and forms SDS-stable oligomeric assembly in the presence of the soluble aqueous extract of curcumin. **(A)** Flow cytometry-based assay showing binding of VCC with human erythrocytes in the presence of the soluble aqueous extract of curcumin in PBS [curcumin-PBS; 80% (v/v)] (red curve). The binding of VCC to the cells in the absence of curcumin extract is shown in black curve. The filled gray curve represents the control cells stained with primary and secondary antibodies, without the VCC treatment. The data shown here is representative of at least three independent experiments. **(B)** VCC retains its ability to form SDS-stable oligomers in the human erythrocytes membrane ghost in the presence of curcumin-PBS [90% (v/v)]. VCC was incubated with human erythrocytes membrane ghost in the presence or absence of curcumin-PBS, and the membrane-bound VCC was probed by pull down-based assay, followed by SDS-PAGE and immunoblotting. Sample treated with SDS-PAGE sample buffer without boiling allowed detection of the SDS-stable oligomers formed by VCC. The data shown here is representative of three independent experiments. **(C)** The ability of VCC to associate and form SDS-stable oligomers in the membrane lipid bilayer of Asolectin-cholesterol liposomes was probed by the pull down-based assay. Liposomes were treated with VCC in the absence or presence of curcumin-PBS [90% (v/v)], and liposome-bound pellet fractions were analyzed by SDS-PAGE and Coomassie staining, with or without boiling in the SDS-PAGE sample buffer. Unboiled samples allowed detection of the SDS-stable oligomers formed by VCC. The bands corresponding to the monomeric and oligomeric form of VCC are indicated with arrow and asterisk, respectively. Lane M shows the molecular weight marker. The data shown here is representative of at least three independent experiments. **(D)** A control experiment was performed to examine whether VCC could form any SDS-stable oligomer in solution in the presence of curcumin extract in PBS, in the absence of the target membranes. VCC (500 nM) was incubated in the absence or presence of the curcumin extract in PBS (90% vol/vol) in a reaction volume of 1 ml in PBS, for 1 h at 25°C. Samples (40 μl) were withdrawn from each reaction mixture, divided into two equal parts, and mixed with SDS-PAGE sample buffer. One part was boiled, while the other part was incubated at room temperature. Unboiled and boiled samples were analyzed by SDS-PAGE/Coomassie staining. The bands corresponding to the monomeric form of VCC are indicated with an arrow. Lane M shows the molecular weight marker. The data shown here is representative of three independent experiments. The result showed that VCC did not form any SDS-stable oligomer in solution in the presence of curcumin extract in PBS, in the absence of the target membranes.

Our result described above showed that the pore-forming activity of VCC against the target membranes was compromised in the presence of the soluble aqueous extract of curcumin in PBS (curcumin-PBS extract). Therefore, we examined whether VCC could interact with the target membranes in the presence of the soluble aqueous extract of curcumin (curcumin-PBS extract). Flow cytometry-based assay of binding showed that the presence of curcumin-PBS did not affect the binding of VCC toward the cell surface of human erythrocytes (Figure 4A). Pull down-based assay also confirmed that the binding of VCC to the human erythrocytes membrane ghost and Asolectin-cholesterol liposomes, and its ability to form SDS-stable oligomers were not affected to any noticeable extent in the presence of curcumin-PBS (Figures 4B,C). It is important to mention that VCC did not form any SDS-stable oligomer in solution in the presence of curcumin extract in PBS, in the absence of the target membranes (Figure 4D). Altogether, these results suggest that the soluble extract of curcumin in the aqueous medium interferes with the membrane-damaging action of VCC through a different mechanism, as compared to that imposed by the insoluble fraction of curcumin. VCC can bind to the target membranes and form the oligomeric assembly in the presence of the soluble extract of curcumin. However, it appears from our data that such oligomeric assembly of VCC in the target membranes, generated in the presence of the soluble extract of curcumin, cannot exert the membrane-damaging activity. It remains unclear how exactly the soluble extract of curcumin arrests the oligomeric form of VCC in an abortive state and compromises the functional pore-formation by the membrane-bound fraction of the toxin.

### Docking of Curcumin Onto *Vibrio cholerae* Cytolysin Predicts Potential Binding Site(s)

To identify the most likely binding site(s) of curcumin, we predicted the potential binding pockets or cavities on the surface of VCC and docked curcumin on these sites. We predicted pockets using the methods, DeepSite, P2rank, and CurPocket. DeepSite predicted five pocket centers, and from P2rank and CurPocket, we extracted the top 20 ranked pockets. Further, these pockets centers were clustered to obtain the consensus sites (Supplementary Table 1). As observed in Supplementary Figure 3, three consensus sites (1–3) were predicted in all the methods. However, the rest other six consensus sites consisting of pockets were identified by at least two of the three methods.

**FIGURE 5:**
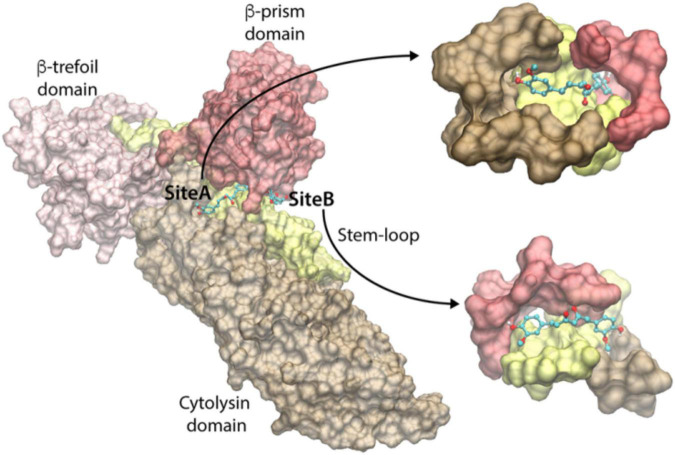
Predicted curcumin-binding sites of VCC. The close-up views of the binding cavities of site-A and site-B are shown with the docked conformation of curcumin. VCC structure is represented by transparent Surf representation. Cytolysin domain, β-trefoil domain, pre-stem motif, and β-prism domain are shown in brown, pink, yellow, and red colors, respectively. The docked curcumin in site-A and site-B is represented as the ball-and-stick model, with carbon, oxygen, and hydrogen atoms shown in cyan, red, and white colors, respectively.

To identify the curcumin-binding site(s) among these predicted pockets, we docked curcumin (keto-form) on all the pockets using the docking program Vina. The docking energy scores of the best pose for the consensus sites are shown in Supplementary Table 1. We observed that the docking on the pockets of the three consensus sites 2, 8, and 9 resulted in the highest curcumin-docked energy score of −8.0 kcal/mol. Interestingly, the closer examination of the docking poses revealed that the curcumin-docked poses are spatially similar in the consensus sites 8 and 9. However, this docked pose is located distantly away from the docked curcumin pose of site 2 (Figure 5). Thus, we have identified two potential curcumin-binding sites (referred to as site-A and site-B) having the same docking energy score (Figure 5). We analyzed the protein-ligand interactions using the LigPlot+ and visually examined the shape of the cavities. As can be seen in Figure 5, the site-A binding cavity is deep and forms an enclosed shape for the ligand binding, whereas the site-B cavity is shallow. Based on the shape of the cavity, it is quite likely that site-A may be able to bind curcumin more efficiently. The site-A cavity lies at the interface of the three domains/motif of VCC (cytolysin domain, β-prism domain, and pre-stem motif of the cytolysin domain), and residues from these modules contribute to curcumin-binding (Supplementary Figure 4A). In contrast, site-B lies predominantly at the interface of the pre-stem motif and the β-prism domain (Supplementary Figure 4B). It is important to note that mostly the hydrophobic interactions dominate the binding of curcumin and VCC. Overall, the docking and subsequent analyses predict that both site-A and site-B can potentially bind curcumin. High-resolution structural studies targeting the VCC-curcumin complex would provide exact structural details regarding the roles of these two predicted sites in curcumin-binding.

## Conclusion

Our study, for the first time, reveals that curcumin has the potential to act as the natural inhibitor for the membrane-damaging pore-forming action of VCC. Our results suggest that curcumin can directly interact with VCC and can interfere with its membrane-damaging function. The mechanism by which curcumin inhibits VCC functionality appears to depend on the mode of curcumin preparation. VCC can associate with the insoluble fraction of curcumin present in the aqueous environment and thus separates from the solution phase. This, in turn, reduces the availability of VCC in solution to exert its action on the target membranes. In contrast, the soluble extract of curcumin in the aqueous medium does not interfere with the membrane-binding step of VCC; instead, it appears to compromise the functional pore-formation by the membrane-bound fraction of the toxin. Previous studies have shown that curcumin can compromise the mode of action of two prominent bacterial β-PFTs, LLO and *S. aureus* α-hemolysin, presumably by affecting the oligomerization efficacy of these proteins (Wang et al., 2016; Zhou et al., 2017). LLO is a large pore-forming β-PFT (Koster et al., 2014), and its structural disposition is markedly different from that of VCC. Consistent with this, curcumin appears to bind to distinctly different regions in these two toxins. In the case of LLO, curcumin binds to a pocket located between the domain 2 and domain 4 of the toxin (Zhou et al., 2017). In contrast, as predicted in our present study, curcumin can bind to the different location(s) in the VCC structure. Therefore, it is not surprising that curcumin affects the functions of LLO and VCC through the different mechanism. *S. aureus* α-hemolysin and VCC are more closely related in terms of their structure, and they also belong to the family of the small pore-forming β-PFTs. As predicted from our present study, curcumin appears to bind to two possible sites on VCC, both of which involve the pore-forming pre-stem motif. Interestingly, one previous study has shown that curcumin can bind to the pore-forming stem region of α-hemolysin, and blocks deoxycholate-induced oligomerization of α-hemolysin (Wang et al., 2016). However, effect of curcumin on the oligomerization process of α-hemolysin in the membrane lipid bilayer has not been explored in that study. Nevertheless, the exact mechanism by which curcumin can exert its inhibitory effect on a specific PFT would possibly vary depending on the exact nature of the PFT and the mode of its interaction with curcumin. More future studies would be required to explore and address this notion in detail. Altogether, our study presents curcumin as a potent agent to neutralize the membrane-damaging effect of VCC. Further exploration in this direction would be required to examine whether curcumin can exert a similar inhibitory effect on the broad range of PFTs from diverse pathogenic bacteria.

## Data Availability Statement

The original contributions presented in the study are included in the article/Supplementary Material. Further inquiries can be directed to the corresponding author.

## Ethics Statement

The studies involving human participants were reviewed and approved by Institute Ethics Committee of Indian Institute of Science Education and Research Mohali. The participants provided their written informed consent to participate in this study.

## Author Contributions

MS performed research and wrote the manuscript. NR and SP performed the computational analysis and wrote the corresponding section of the manuscript. KC supervised the overall research and wrote and prepared the final version of the manuscript. All authors contributed to the article and approved the submitted version.

## Conflict of Interest

The authors declare that the research was conducted in the absence of any commercial or financial relationships that could be construed as a potential conflict of interest.

## Publisher’s Note

All claims expressed in this article are solely those of the authors and do not necessarily represent those of their affiliated organizations, or those of the publisher, the editors and the reviewers. Any product that may be evaluated in this article, or claim that may be made by its manufacturer, is not guaranteed or endorsed by the publisher.

## Abbreviations

PFT: pore-forming toxin
VCC: *Vibrio cholerae* cytolysin.
