# Supplementary material for: Curcumin Inhibits Membrane-Damaging Pore-Forming Function of the β-Barrel Pore-Forming Toxin Vibrio cholerae Cytolysin
Source: Front Microbiol. 2022 Jan 24;12:809782. doi: 10.3389/fmicb.2021.809782 (PMC8818996; doi:10.3389/fmicb.2021.809782)
Supplement: Supplementary file 1 [file Data_Sheet_1.docx]

**Supplementary Material**

**Curcumin inhibits membrane-damaging pore-forming function of the β-barrel pore-forming toxin *Vibrio cholerae* cytolysin**

Running title: Curcumin inhibits pore-forming toxin VCC

**Mahendra Singh, N Rupesh, Shashi Bhushan Pandit, Kausik Chattopadhyay***

Department of Biological Sciences,

Indian Institute of Science Education and Research Mohali

Sector 81, SAS Nagar, Manauli, Mohali, Punjab 140306, India

***Corresponding author:**

Dr. Kausik Chattopadhyay

Department of Biological Sciences

Indian Institute of Science Education and Research Mohali

Sector 81, SAS Nagar, Manauli, Mohali, Punjab 140306, India

Tel: 91-0172-2293147; Fax: 91-0172-2240124

E-mail: [kausik@iisermohali.ac.in](about:blank)

ORCID: 0000-0001-8529-9475

**Table S1. Summary of the consensus predicted pockets and docking energy score**

| **Consensus pocket number** | **Pocket id from various predictors*** | **Best docking energy score** |
| --- | --- | --- |
| Csite-1 | Pocket-D1, pocket-P1, pocket-C4 | -7.0 |
| Csite-2 | Pocket-D2, pocket-P2, pocket-C2 | -8.0 |
| Csite-3 | Pocket-D3, pocket-P19, pocket-C9 | -6.6 |
| Csite-4 | Pocket-D4, pocket-P11 | -6.2 |
| Csite-5 | Pocket-D5, pocket-C19 | -6.1 |
| Csite-6 | Pocket-P4, pocket-C3 | -7.5 |
| Csite-7 | Pocket-P9, pocket-C10 | -6.1 |
| Csite-8 | Pocket-P12, pocket-C15 | -8.0 |
| Csite-9 | Pocket-P17, pocket-C16 | -8.0 |

*predicted pockets are assigned identifier based on the method used for the prediction and the rank of the pocket. The method DeepSite, P2rank, and CurPocket are abbreviated as D, P, and C, respectively.

**
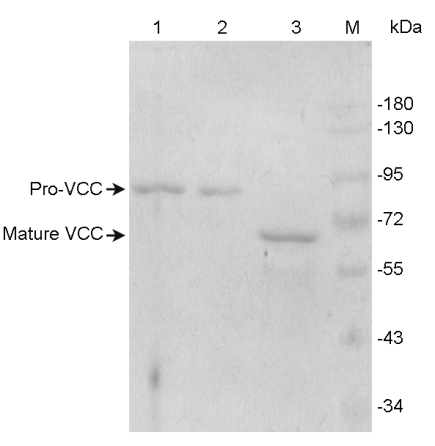
**

**Figure S1. SDS-PAGE and Coomassie staing profile of VCC at the different stages of purification.** Lane 1, Pro-VCC obtained after Ni-NTA Agarose affinity chromatography; Lane 2, purified form of Pro-VCC after ion-exchange chromatography on Q Sepharose Fast Flow resin; Lane 3, purified form of mature VCC after the ion-exchange chromatography on Q Sepharose Fast Flow resin. Lane M shows the molecular weight markers.

**
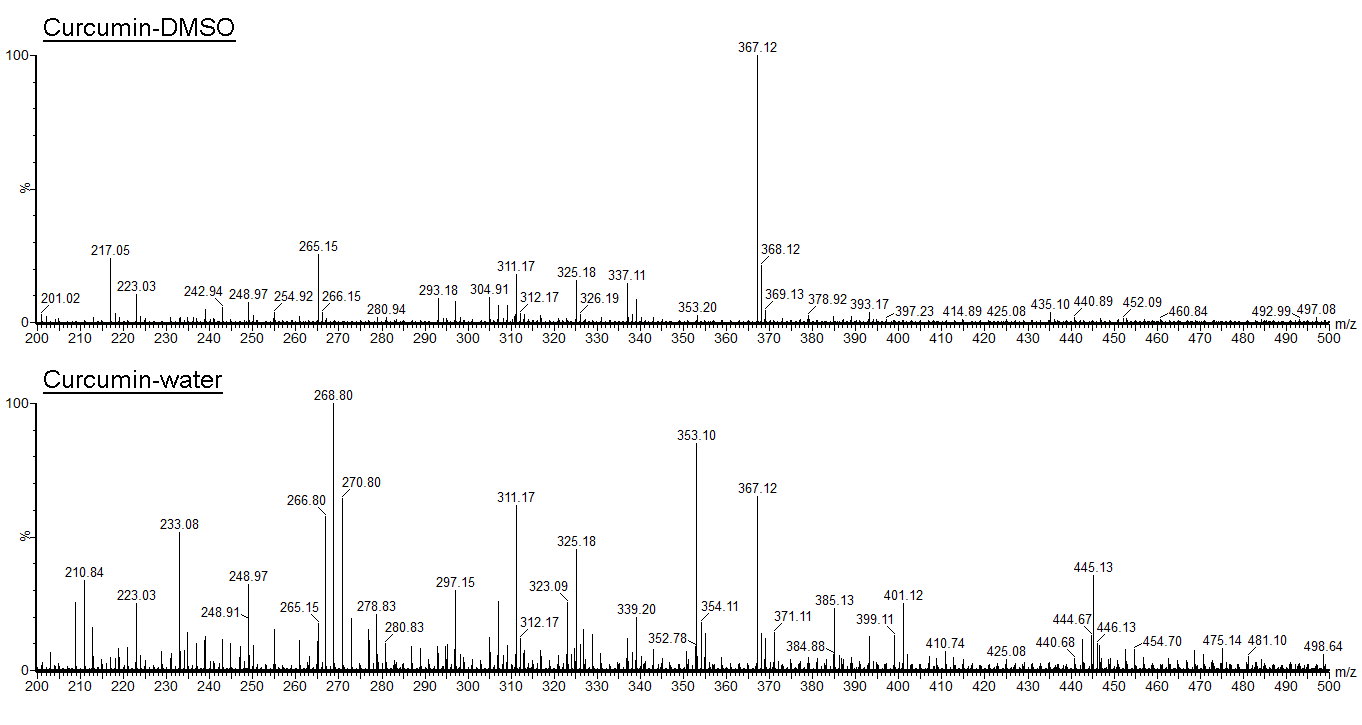
**

**Figure S2. Mass spectrometry profiles of the curcumin-DMSO preparation, and the soluble aqueous extract of curcumin in water (curcumin-water) generated upon heat treatment.**

**
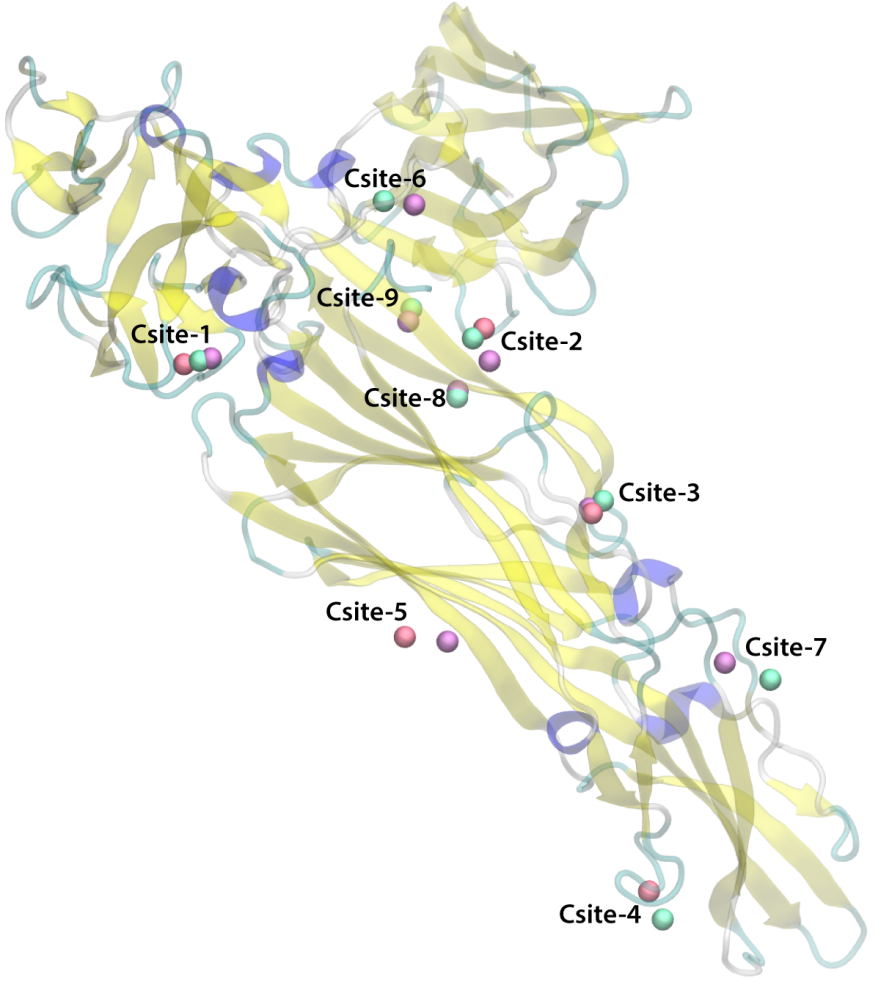
**

**Figure S3. VCC structure showing the consensus of the predicted pockets from DeepSite, P2rank, and CurPocket.** The protein secondary structures, helices, sheets, and loops are shown in blue, yellow and cyan colors, respectively. The pocket centers predicted from DeepSite, P2rank and CurPocket are represented as sphere in red, green, and purple colors, respectively.

**
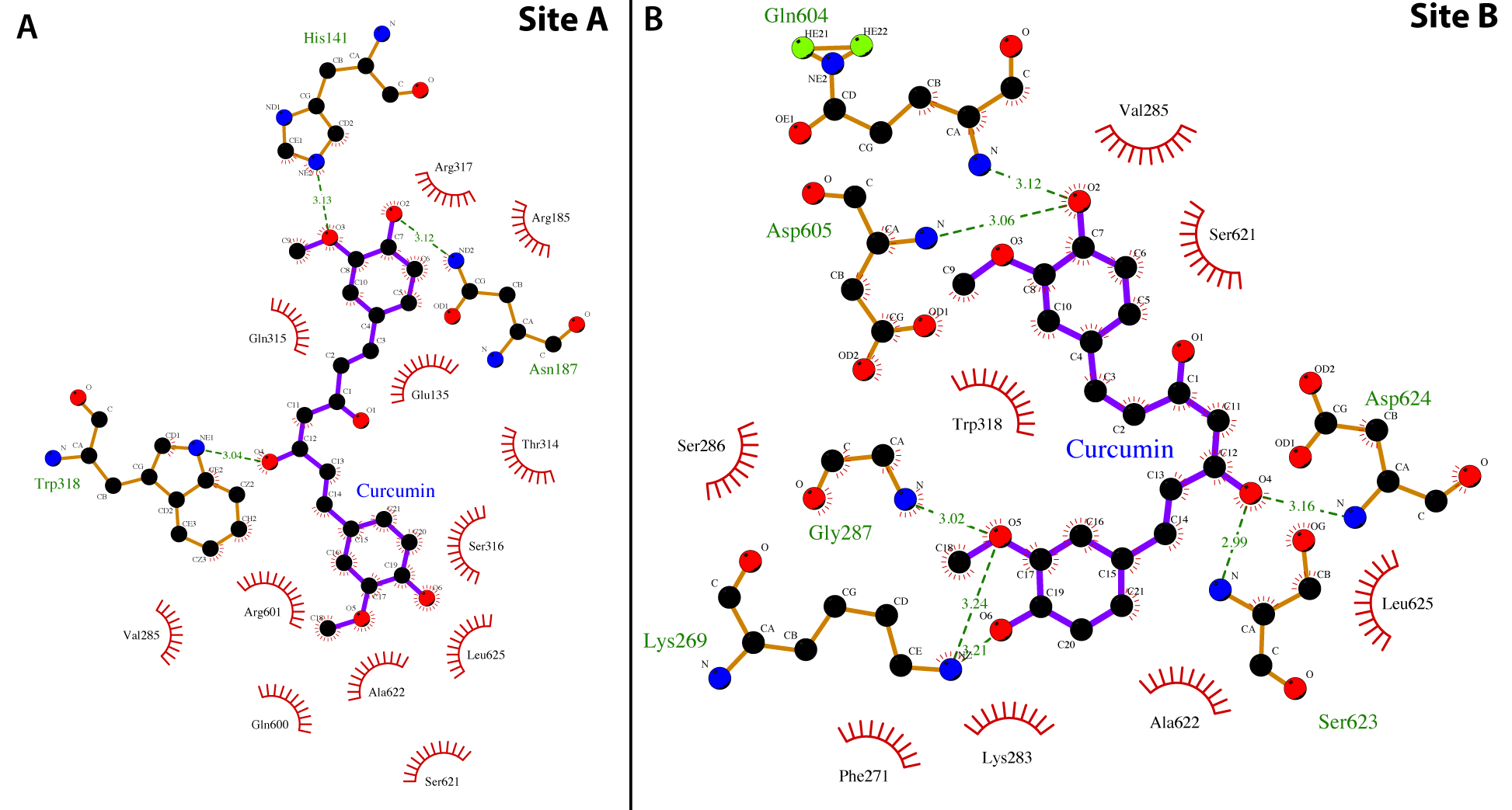
**

**Figure S4. The best-docked pose of curcumin at site-A and site-B onto the VCC structure.** The figure shows the LigPlot+ representation of the VCC-curcumin interactions. Potential hydrophobic interactions are depicted with the ray representation, and the putative hydrogen bonds are shown with dotted green line.
